# Functional Quality, Mineral Composition and Biomass Production in Hydroponic Spiny Chicory (*Cichorium spinosum* L.) Are Modulated Interactively by Ecotype, Salinity and Nitrogen Supply

**DOI:** 10.3389/fpls.2019.01040

**Published:** 2019-08-30

**Authors:** Martina Chatzigianni, Georgia Ntatsi, Maria Theodorou, Aristidis Stamatakis, Ioannis Livieratos, Youssef Rouphael, Dimitrios Savvas

**Affiliations:** ^1^Department of Crop Science, Laboratory of Vegetable Crops, Agricultural University of Athens, Athens, Greece; ^2^Department of Sustainable Agriculture, Laboratory of Soil Science and Plant Diagnostics, Mediterranean Agronomic Institute of Chania, Chania, Greece; ^3^Institute of Plant Breeding and Genetic Resources ELGO-DEMETER, Thessaloniki, Greece; ^4^Department of Agricultural Sciences, University of Naples Federico II, Portici, Italy

**Keywords:** bioactive molecules, closed soilless system, landraces, macro-minerals, nitrate, salinity eustress, stamnagathi

## Abstract

The hydroponic cultivation of spiny chicory (*Cichorium spinosum* L.), also known as stamnagathi, allows the development of year-round production. In the current study, two contrasting stamnagathi ecotypes originating from a montane and a coastal-marine habitat were supplied with nutrient solution containing 4 or 16 mM total-N in combination with 0.3, 20, or 40 mM NaCl. The primary aim of the experiment was to provide insight into salinity tolerance and nutrient needs in the two ecotypes, thereby contributing to breeding of more resilient cultivars to salinity and nutrient stress. Nutritional qualities of the stamnagathi genotypes were also tested. The coastal-marine ecotype was more salt tolerant in terms of fresh shoot biomass production and contained significantly more water and macro- and micro-nutrients in the shoot per dry weight unit. The root Na^+^ concentration was markedly lower in the coastal-marine compared to the montane ecotype. The leaf Na^+^ concentration was similar in both ecotypes at external NaCl concentrations up to 20 mM, but significantly higher in the montane compared to the coastal-marine ecotype at 40 mM NaCl. However, the leaf Cl^−^ concentration was consistently higher in the coastal-marine than in the montane ecotype within each salinity level. The marine ecotype also exhibited significantly less total phenols, carotenoids, flavonoids, and chlorophyll compared to the montane ecotype across all treatments. Integrating all findings, it appears that at moderate salinity levels (20 mM), the higher salt tolerance of the coastal-marine ecotype is associated with mechanisms mitigating Na^+^ and Cl^−^ toxicity within the leaf tissues, such as salt dilution imposed through increased leaf succulence. Nevertheless, at high external NaCl levels, Na^+^ exclusion may also contribute to enhanced salt tolerance of stamnagathi. Both ecotypes exhibited a high N-use efficiency, as their shoot biomass was not restricted when the total-N supply varied from 16 to 4 mM. The leaf organic-N was not influenced by salinity, while the interaction ecotype × N-supply-level was insignificant, indicating that the mechanisms involved in the salt tolerance difference between the two ecotypes was not linked with N-acquisition or -assimilation within the plant. The current results indicate that both ecotypes are promising germplasm resources for future breeding programs.

## Introduction

Consumer perception of the capacities of fresh functional plant-based foods to support human health and longevity has increased, especially during the last two decades (Kyriacou et al., 2016). These changes in consumer behavior have fueled the critical reassessment of the fresh-fruit and -vegetable qualities as part of the ‘personalised nutrition’ concept, as defined in a recent paper as ‘*a dynamic composite of physicochemical properties and evolving consumer perception, which embraces organoleptic, nutritional and bioactive components*’ (Kyriacou and Rouphael, 2018). Accordingly, crop growers, food nutritionists, extension specialists, as well as scientists are seeking to identify vegetable crops that may be produced inexpensively for the fresh-food markets and that present high nutraceutical (and organoleptic) properties (Slavin and Lloyd, 2012).

Towards that end, spiny chicory (*Cichorium spinosum* L.), a dwarf perennial species within the *Asteraceae* family, which is also known in Greek language as *stamnagathi*, is gaining popularity in many parts of Greece (Crete) and throughout the Mediterranean basin (Cyprus, Libya, Malta, Sicily, Spain, and Turkey) as a functional culinary trend (Brieudes et al., 2016). In addition to its unique taste, consumption of *C. spinosum* has increased to its high levels of health-promoting phytochemical compounds, such as vitamins (C, E, and K1), phenolic acids (chicoric and 5-O-caffeoylquinic), total glutathione, proteins, fatty acids, carotenoids (β-carotene and lutein), and minerals (Zeghichi et al., 2003; Vardavas et al., 2006; Petropoulos et al., 2016; Petropoulos et al., 2017). The commodity commands very high prices and is ever increasingly demanded by fresh vegetable markets. Therefore, the greenhouse cultivation of *C. spinosum* has the immediate potential for expansion (Ntatsi et al., 2017a). However, in many areas of the Mediterranean region, vegetable farmers are forced to use poor quality water (i.e., highly saline), and especially in coastal-marine regions where leafy vegetables are grown under protected cultivation. Excessive concentration of sodium chloride (NaCl) in irrigation water and/or soil induces osmotic as well as ionic stresses, which lead to several morphological, anatomical, physiological, and metabolomic changes (Munns, 2005; Colla et al., 2013a; Ntatsi et al., 2017a; Rouphael et al., 2017a; Rouphael et al., 2017b; Rouphael et al., 2018a). In particular, the excessive Na^+^ and Cl^−^ concentrations in the root zone are harmful to most vegetable crops, as they can cause pigment (chlorophyll and carotenoids) degradation (Lucini et al., 2015), hamper the macro- and micronutrient uptake, translocation, and assimilation (Grattan and Grieve, 1998), and limit net CO_2_ assimilation (Colla et al., 2013b). As a result, the plants exhibit stunted growth and yield is reduced significantly (Lucini et al., 2016; Rouphael et al., 2017a). Although salinity generally reduces the crop productivity, in many cases mild to moderate salt stress also known as *eustress* can trigger the biosynthesis and accumulation of bioactive secondary compounds (carotenoids, phenolic compounds, organosulfuric compounds, polyamines, *etc.*), as demonstrated for several vegetable crops grown under protected cultivation (Rouphael et al., 2012, Rouphael et al., 2017a; Rouphael et al., 2017b; Rouphael et al., 2018b). However, the accumulation or degradation of specific organic molecules and secondary metabolites depends on several interacting factors such as plant species or ecotype (cultivar), and the period and magnitude of exposure, as well as the agronomic management options (Rouphael and Kyriacou, 2018).

Moreover, leafy vegetables grown commercially, particularly those cultivated in hydroponics, are focused on maximizing yield through intensification of fertilizer use and especially nitrates, the most important source of nitrogen (N), which boosts productivity (Borgognone et al., 2013). Minimizing N supply, while maintaining yield and nutritional qualities and avoiding negative environmental impacts, is of special importance to growers and presents a major sustainability challenge to the vegetable industry (Colla et al., 2010; Colla et al., 2011; Borgognone et al., 2016). In addition, several authors (Stefanelli et al., 2010; Becker et al., 2015; Borgognone et al., 2016) have demonstrated that low N-availability can increase the concentration of health promoting bioactives such as phenolic acids and flavonoids, as shown for several diverse forms of leafy vegetable crops destined for fresh consumption as well as for the food-processing industries.

Despite the increasing economic importance of *C. spinosum* as a new niche product, there is a lack of information in the scientific literature concerning its response to salt stress. One relevant study is that of Klados and Tzortzakis (2014) who found that increasing the NaCl concentration in the supplied nutrient solution to 40 mmol L^−1^ reduced the plant biomass production but increased the concentration of total phenolics, bitterness, and sourness of leaves. In another study, Petropoulos et al. (2017) found that increasing the electrical conductivity to 6 and 8 dS m^−1^ in the root zone of *C. spinosum* increased the leaf protein content on fresh weight basis, and the antioxidant activity, but had no impact on the concentration of phenolic compounds. Nevertheless, nothing is known regarding how levels of these phytochemicals vary in response to N-fertilizer dose, NaCl concentration, or their interaction.

Considering this background, a three-factorial experiment was designed to study the effects of N level and NaCl concentration in the supplied nutrient solution on growth, mineral composition, and nutritional attributes (phenolics, carotenoids, antioxidant activity, and flavonoids) of two contrasting stamnagathi ecotypes originating either from a coastal-marine or from a montane habitat. The primary aim of this experiment was to provide some insight into the mechanisms underlying salinity tolerance of these two contrasting ecotypes, as well as into their possible links to the level of N supply, thereby contributing to breeding of more resilient cultivars to salinity and nutrient stress. An additional objective of the present study was to test the hypothesis that moderate levels of combined salinity and N-shortage stress improve some nutritional quality characteristics of stamnagathi without compromising fresh biomass production, which is of economic interest for growers.

## Materials and Methods

### Growth Conditions and Plant Material

Seeds of stamnagathi (*Cichorium spinosum* L.) were harvested from wild plants originating from two different areas of Crete: a coastal zone (Stavros, North-East Chania Crete, 35°59′17.79″N and 24°09′79.74″E) and a montane site (Tavri at Omalos on the mountain Lefka Ori in Chania, 1200 m altitude, 35°29′30.77″N, 24°15′78.44″E). The collection of the stamnagathi seeds took place on 23^rd^ and 25^th^ of September 2014 for the montane and coastal-marine ecotypes, respectively. The seeds were germinated as described below, and the resultant seedlings were cultivated in an unheated glasshouse during the 2015 winter–spring growing season at the Mediterranean Agronomic Institute of Chania (MAICh) Crete, Greece (35°29′40.45″N and 24° 02′57.42″E). The glasshouse layout of the test material comprised five double-rows of each ecotype, plus one additional row at each outer-border that served as a guard-row. The length and width of each double-row were 10 and 0.2 m, respectively. The plants were grown under natural light conditions. The average day/night temperatures (± standard deviations) inside the glasshouse were 19.34 ± 2.15/12.64 ± 1.68°C in February, 21.39 ± 2.03/14.47 ± 1.73°C in March, 23.65 ± 2.05/16.03 ± 2.04°C in April, and 25.69 ± 0.84/17.83 ± 1.39°C in May (from the 1^st^ to the 18^th^).

### Experimental Design, Crop, and Nutrient Solution Management

On 4^th^ of December 2014, the seeds of the two ecotypes were first sown in trays, with one seed being placed into one of the 84 holes (per tray). Each hole was filled with a peat:perlite (3:1 [v/v]) mixture. The pH of the growing medium was 6 and no NPK fertilizers were added, as the peat was enriched with nutrients. The macronutrient content of peat was as follows: Nitrogen, phosphorus, potassium, and magnesium were 100, 115, 125, and 100 mg L^−1^, respectively, while all essential trace elements were included (Klassman Plug Mix extra plus). For that reason, the seedlings were irrigated with water regularly as required, while all treatments were applied after transplanting. On 30^th^ of January 2015, 2 months after sowing, the seedling-plugs were removed from the tray and transplanted into perlite packed in bags (Perloflor Hydro 1, Athens, Greece). The particle size of the perlite granules ranged from 0.5 to 2.5 mm. After transplanting, the plants were fertigated using different nutrient solutions in each treatment as described below, and the drainage water was not reused. Each perlite bag (33 L) was 1 m in length, 24 cm in width, and 16 cm in height and accommodated four equally spaced plants. The space between plants within rows was 20 cm, with 48 plants per row. To allow free drainage of excess nutrient solution, two holes were made at the bottom of each bag.

Twelve treatments were derived from a factorial combination of three NaCl concentrations (0.3, 20, or 40 mM), two levels of -N concentrations (4 or 16 mM), and two stamnagathi ecotypes from either a montane (M) or a coastal (C) site. The treatments were arranged in a randomised complete-block design with four replicates per treatment. As a total, 48 experimental units (plots) with 12 plants (3 bags × 4 plants per bag) in each plot (n = 576 plants) were established. The salinity and N treatments were initiated directly after transplanting.

The macronutrient concentrations in the different nutrient solution treatments are presented in **Table 1**. The micronutrient concentrations were identical in all treatments, as follows: 15.0 µM Fe, 8.0 µM Mn, 6.0 µM Zn, 0.7 µM Cu, 30.0 µM B, and 0.5 µM Mo. The decrease of the NO_3_-N level from 16 to 4 mM in the low NO_3_-supply treatments was compensated for by an equivalent increase of the SO_4_^2−^ and Cl^−^ concentrations, thereby maintaining the same total nutrient anion and cation concentrations in both NO_3_-supply levels. Thus, the only differences in EC between treatments were those imposed by the addition of NaCl. The pH of the nutrient solution supplied to the plants was 5.6 in all treatments, while the EC was 2.10, 4.10, and 6.14 dS m^−1^, corresponding to 0.3 mM (non-salt control), 20 mM, or 40 mM NaCl, respectively. Nutrient solution was delivered to the plants through a drip-irrigation system using pumps connected to an electronic timer. Each plant was supplied with nutrient solution from an individual emitter having a flow rate of 2 L h^−1^. The irrigation frequency was adjusted according to the integral of solar radiation intensity aiming to result in a drainage fraction of 30%. This resulted in two to four irrigation applications per day (140–280 ml per plant) to each experimental unit. Moreover, the pH and EC of the drainage solution were monitored during the growing cycle by collecting samples three times per week. During the whole experiment, no spraying for disease or insect control, and no heating was applied.

**Table 1:** The electrical conductivity (EC), pH, and the concentrations of K^+^, Ca^2+^, Mg^2+^, Na^+^, Cl-, NO_3_^−^, H_2_PO_4_^−^, and SO_4_^2−^ in the six different nutrient solution treatments that were applied to the plants by combining two levels of total-N supply (4 or 16 mmol L^−1^, denoted as 4TN and 16TN, respectively) and three different salinity levels (0.3, 20, and 40 mM, respectively) in the nutrient solution.

Salinity	0.3	20	40	0.3	20	40
Total-N level	4 mmol L^−1^			16 mmol L^−1^		
EC (dS m^−1^)	2.10	4.10	6.14	2.10	4.10	6.14
pH	5.60	5.60	5.60	5.60	5.60	5.60
K^+^ (mmol L^−1^)	7.50	7.50	7.50	7.50	7.50	7.50
Ca^2+^ (mmol L^−1^)	4.40	4.40	4.40	4.40	4.40	4.40
Mg^2+^ (mmol L^−1^)	1.50	1.50	1.50	1.50	1.50	1.50
Na^+^ (mmol L^−1^)	0.3	20.00	40.00	0.3	20.00	40.00
Cl^−^ (mmol L^−1^)	6.37	26.07	46.07	0.30	20.00	40.00
NO_3_^−^ (mmol L^−1^)	3.00	3.00	3.00	15.00	15.00	15.00
H_2_PO_4_ (mmol L^−1^)	1.20	1.20	1.20	1.20	1.20	1.20
SO_4_^2−^ (mmol L^−1^)	4.81	4.81	4.81	1.84	1.84	1.84

### Plant Material

The shoots of the plants were harvested on 03/31/2015, i.e., 60 days after transplanting (DAT). The edible shoots were harvested when the majority of non-salinized plants treated with 16 mM were considered commercially ripe according to local cultivation practices (rosette diameter between 20 and 27 cm, number of leaves >30). Harvesting entailed cutting the main stem of each plant at about 1 cm above the growing medium using a sharp knife. After the first production cycle, the roots with the basal part of the stem were left to sprout again for a second production cycle. The second harvest took place on 05/18/2015 (108 DAT). At the second harvest, both the shoots and root of the plants were collected. At both production cycles, the harvested plants from each experimental unit were pooled into a single sample, placed immediately in plastic bags, and transported to the laboratory for processing and further analysis.

### Ionomic Analyses

Leaf samples originating from 12 plants per experimental unit were collected at the first harvest, i.e., 60 days after transplanting, and divided into two sub-samples. The first sub-sample was dried and milled to determine the leaf N, P, K^+^, Ca^2+^, Mg^2+^, and S concentrations. The second sub-sample was stored immediately after harvest at −18ºC and used later to determine total chlorophyll, total phenolics, flavonoids, and β-carotene concentrations, as well as total antioxidant activity. The plant samples collected at the second harvest (2^nd^ production cycle) were separated into shoots and roots and used to determine the concentrations of N, P, K^+^, Ca^2+^, and Mg^2+^ in the roots, Cl^−^ in leaves, and Fe, Mn, Zn, Cu, and Na^+^ in both leaves and roots. The Cl^−^ concentration in the root was not measured, because the samples were lost due to a technical failure. The samples used to determine tissue mineral concentrations were weighed and dried at 80°C for 72 h until they reached a constant weight. Subsequently, they were weighed again to determine the corresponding dry biomass as well as the dry matter percentage (and moisture content). The dried tissue samples (leaves and roots) were powdered using a blade-mill and passed through a 40-mesh sieve. Sub-samples (300 mg) of all dried plant tissue samples were used to determine the concentrations of the above-mentioned macro- and microelements. Organic-N was determined by applying the Kjeldahl method. Briefly, 250 mg of dry leaf and root tissues was digested in a Gerhardt apparatus after adding 10 ml of concentrated H_2_SO_4_ in the presence of a catalyst mixture (100 g K_2_SO_4_ + 16 g CuSO_4_ + 1.5 g Se). The distillation was carried out using a Vapodest 40 (Gerhardt) after addition of 50 ml H_2_O and 60 ml of 40% NaOH (Bremner, 1965). Phosphorus, K^+^, Ca^2+^, Mg^2+^, Na^+^, Mn, Fe, Zn, and Cu were determined in aqueous extracts obtained as follows. Powdered plant tissues placed on crucibles were dry-ashed at 450°C for 6 h, and the ash was dissolved using 10 ml of 2 N hydrochloric acid (HCl). Subsequently, the crucibles were transferred to a hot plate at 80ºC for a few minutes, and the hydrochloric solution was filtered and transferred to 100-ml flasks, which were filled up with distilled water. The mineral concentrations in the obtained aqueous extracts were determined using an Inductively Coupled Plasma-Optical Emission Spectrometry Instrument (ICP-OES PSFO 2.0, Leeman Labs INC., USA). The ICP-OES operating conditions were as follows: nebulizer gas flow rates: 0.5 L min^−1^; auxiliary gas flow: 0.5 L min^−1^; plasma gas flow: 15 L min^−1^; pump rate: 45 rpm, and ICP RF power: 1100 W. The S concentration was extracted from 250 mg samples with deionized water at 80°C in a shaking water bath for 10 min (ShakeTemp SW22, Julabo, Seelbach, Germany). The resulting solution was filtered, diluted, and analyzed by ion chromatography (ICS-3000, Dionex, Sunnyvale, CA, USA). A conductivity detector with IonPac AG11-HC guard column and IonPac AS11-HC analytical column (Dionex Corporation) was used for the analysis of S. The determination of Cl^−^ in the plant-tissue extracts and nutrient solutions was performed by titration with AgNO_3_ in the presence of K_2_CrO_4_ (Eaton et al., 1995).

### Functional Quality Analyses

For the chlorophyll analysis, 5 g of fresh leaf tissue was collected at the first harvest, i.e., on 03/31/2015, from three plants per replicate. Chlorophyll was extracted by grinding the tissue with a mortar and pestle using ammoniacal acetone. The resulting extracts were centrifuged at 3,000 × g for 15 min. The chlorophyll contents were determined by UV-Vis spectrophotometry (Specord 250, Jena, Germany). The absorbance of the solution was measured at 645, 652, and 663 nm for chlorophyll-a, chlorophyll-b, and total chlorophyll contents, respectively. Formulae and extinction coefficients used for the determination of chlorophyll contents were described by Lichtenhaler and Wellburn (1983).

The total phenolic content in methanolic extracts was determined using the Folin-Ciocalteau procedure (Singleton et al., 1999) with gallic acid as a standard. One gram of fresh tissue from two plants per replicate was extracted with 50% methanol (1:1 methanol:dH_2_O) using mortar and pestle. The mixture was put in falcon tubes filled up to 10 ml, followed by sonication for 15 min. Then, the samples were centrifugated for 15 min at 4^ο^C and 5000 rpm. A 100 μL aliquot of the supernatant was combined with 500 μL of Folin-Ciocalteau’s reagent (Sigma Aldrich Inc, St Louis, MO, USA) and 400 μL of 7.5% sodium carbonate/water (w/v). Absorption was measured after 30 min at 765 nm using a UV–vis spectrophotometer, and the result was expressed as mg gallic acid (Sigma Aldrich Inc., St Louis, MO, USA) per 100 g dry weight. Briefly, 1 g of raw plant material was weighted and transferred in a mortar with 4 ml of the extraction buffer (50 ml methanol 80% + 1.37 ml HCl 37%). Afterwards, the falcons were transferred on an orbital shaker at 200 rpm at room temperature and, after 2 h, were centrifuged for 15 min at 5000 rpm. The supernatant (Solution A) was transferred to a new falcon. Folin-Ciocalteau preparation: 1 ml of Folin-Ciocalteau + 9 ml dH_2_O. In the Solution A (300 μL), 3 × 750 μL of Folin-Ciocalteau was added (Solution B), vortexed for a few seconds, and then incubated for 5 min. In Solution B, 3 × 750 μL of Na_2_CO_3 _was added and then stored in a dark place at room temperature for 90 min. Absorbance of the mixture, blue in color, was determined at 765 nm using water as blank.

The aluminium chloride colorimetric method was used to measure the total flavonoids content of the stamnagathi extracts as described by Zhishen et al. (1999). Briefly, 1 g of fresh tissue from two plants per replicate was extracted and 1 ml aliquot of appropriately diluted sample or standard solutions of catechin (20, 40, 60, 80, and 100 mg L^−1^) was added to a 10 ml volumetric flask containing 4 ml H_2_O. At time-zero, 0.3 ml 5% NaNO_2_ was added to the flask. After 5 min, 0.3 ml 10% AlCl_3_ was added. At 6 min, 2 ml 1 M NaOH was added to the mixture. Immediately, the reaction flask was diluted to volume with the addition of 2.4 ml of H_2_O and thoroughly mixed. Absorbance of the mixture, pink in color, was determined at 510 nm using water as blank. Total flavonoids of leaves were expressed on a fresh weight basis as mg 100 g^−1^ catechin equivalents (CE).

The total antioxidant activity was determined according to the DPPH (2,2-diphenyl-L-picrylhydrazyl) radical scavenging spectrophotometric assay as reported by Choi et al. (2002). For this analysis, 10 ml of methanol (80%) was mixed in a falcon tube with 1 g of fresh tissue obtained from two plants per replicate and transferred on an orbital shaker for 2 h at 200 rpm. The fresh tissue was collected from a leaf originating from the middle of the rosette, taking care to be of the same physiological age in all samples. Subsequently, the falcons were centrifuged for 15 min at 5000 rpm and the supernatant was transferred to a new 50 ml falcon. For the preparation of the DPPH solution, 2.36 mg DPPH was diluted in 100 ml MeOH. Afterwards, 25 μL of supernatant and 975 μL DPPH solution were vortexed and stored in a dark place for 30 min (t = 30). Absorbance of the mixture was recorded at 515 nm, using methanol as blank (t = 0).

Finally, carotenoids (β-carotene) were determined according to the Nagata and Yamashita (1992) method. In specific, 1 g of fresh cut leaves from two plants per replicate was homogenized with acetone:hexane 4:6 [v:v] using a mortar and a pestle. The mixture was placed in 50 ml falcons, filled up to the final volume of 16 ml with acetone:hexane mixture, shaken vigorously, and stored overnight until the two phases were separated. An aliquot was taken from the upper solution for measurement of optical density at 663, 645, 505, and 453 nm in spectrophotometer, using acetone:hexane mixture as blank. Carotenoids were calculated according to the following equation: β-caroten*e* = 0.216*A_663_−1.22*A_645_−0.304*A_505_+0.452*A_453_ and expressed as mg 100 ml^−1^ of extract.

### Statistical Analysis

Experimental data were analysed by applying three-way ANOVA to assess main effects [Ecotype (E), Total-N, and Salinity], three first-order interactions (E × total-N, E × Salinity, Total-N × Salinity), and one second-order interaction (E × total-N × Salinity). Multiple comparisons of means were performed by applying Duncan’s Multiple Range Test at a confidence level of 0.05 after performing three-way ANOVA. All statistical analyses were carried out using the STATISTICA software package, version 9.0 for Windows (StatSoft Inc., Tulsa, USA). The normality was respected for all parameters, and no data transformation was required for any parameter.

## Results

### Biomass and Yield Production

The shoot fresh weight (FW) of both stamnagathi ecotypes was not influenced by the level of total-N in the supplied nutrient solution, and no interactions between E × N, N × S, and E × N × S were observed. (**Table 2**). However, an interaction between the ecotype and salinity was observed. In particular, the shoot FW was similar in both ecotypes under non-saline conditions, but was significantly higher in the coastal-marine than in the montane ecotype at moderate (20 mM) and high (40 mM) NaCl-salinity. Furthermore, the shoot DW and the dry matter content (DMC) were significantly higher in the montane ecotype in comparison with the coastal-marine ecotype (**Table 2**). For both ecotypes, the shoot dry weight (DW) was restricted by the increase of salinity from 0.3 to 40 mM, while at 20 mM NaCl the reduction in shoot DW was insignificant. However, the N level applied in the nutrient solution had no significant impact on shoot DW (**Table 2**). Salinity and total-N supply level had no impact on the shoot DMC of stamnagathi.

**Table 2:** Impact of seed origin (montane or coastal-marine ecotype), total nitrogen concentration (4 or16 mmol L^−1^, denoted as 4-N and 16-N, respectively), and salinity (0.3, 20, and 40 mM, respectively) in the nutrient solution supplied to hydroponically grown stamnagathi on shoot fresh weight (FW), shoot dry weight (DW), shoot dry matter content, chlorophyll a (Chl a), chlorophyll b (Chl b), and total chlorophyll (Tot chl).

Sources of variation		Shoot FW (g/plant) 1^st^ growing cycle	Shoot DW (g/plant) 1^st^ growing cycle	Dry matter content (%) 1^st^ growing cycle	Chl a (mg g^−1^ FW) 1^st^ growing cycle	Chl b (mg g^−1^ FW) 1^st^ growing cycle	Tot chl (mg g^−1^ FW) 1^st^ growing cycle
Ecotype	Montane	24.7	3.03	12.00	1.26	0.45	1.78
	Coastal	29.9	2.65	8.28	0.90	0.30	1.26
N level (N)	4 mM	27.7	2.71	10.15	1.03	0.35	1.45
	16 mM	26.8	2.97	10.14	1.12	0.40	1.59
Salinity	0.3 mM	27.4	3.14^a^	10.22	1.04^b′^	0.36	1.45^b′^
	20 mM	27.9	2.81^ab^	10.11	1.10^a′^	0.38	1.56^a^
	40 mM	26.7	2.57^b^	10.11	1.09^ab′^	0.38	1.56^a′^
**Interactions**							
E × N	Montane 4-N	25.4	2.88	11.84	1.22	0.43	1.71
	Montane 16-N	23.9	3.18	12.16	1.30	0.47	1.85
	Coastal 4-N	30.0	2.55	8.45	0.85	0.28	1.19
	Coastal 16-N	29.7	2.75	8.11	0.94	0.32	1.32
E × S	Montane 0.3 NaCl	26.8 ^bc^	3.57	12.08	1.16^b^	0.42	1.63^b^
	Montane 20 NaCl	24.2 ^cd^	2.90	12.02	1.34^a^	0.47	1.89^a^
	Montane 40 NaCl	23.1 ^d^	2.63	11.91	1.28^a^	0.47	1.83^a^
	Coastal 0.3 NaCl	27.9 ^b^	2.71	8.34	0.91^c^	0.31	1.26^c^
	Coastal 20 NaCl	31.5 ^a^	2.72	8.20	0.97^c^	0.29	1.23^c^
	Coastal 40 NaCl	30.2 ^ab^	2.52	8.30	0.90^c^	0.30	1.28^c^
N × S	4 N – 0.3 NaCl	28.9	2.87	10.24	1.03^C^	0.36	1.44^B^
	4 N – 20 NaCl	27.0	2.82	10.10	1.07^BC^	0.35	1.49^B^
	4 N – 40 NaCl	27.3	2.44	10.10	0.99^C^	0.35	1.42^B^
	16 N – 0.3 NaCl	25.8	3.41	10.19	1.04^C^	0.36	1.45^B^
	16 N – 20 NaCl	28.7	2.81	10.12	1.13^AB^	0.41	1.63^A^
	16 N – 40 NaCl	26.0	2.69	10.11	1.19^A^	0.42	1.69^A^
E × N × S	Montane 4 N – 0.3 NaCl	28.7	3.18	11.80	1.18	0.43^b^	1.66
	Montane 4 N – 20 NaCl	24.2	2.90	11.97	1.30	0.42^b^	1.81
	Montane 4 N – 40 NaCl	23.4	2.57	11.74	1.17	0.43^b^	1.66
	Montane 16 N – 0.3 NaCl	24.8	3.96	12.36	1.13	0.40^b^	1.60
	Montane 16 N – 20 NaCl	24.2	2.91	12.06	1.37	0.51^a^	1.96
	Montane 16 N – 40 NaCl	22.8	2.68	12.07	1.40	0.52^a^	2.00
	Coastal 4 N – 0.3 NaCl	29.0	2.56	8.67	0.88	0.29^cd^	1.22
	Coastal 4 N – 20 NaCl	29.8	2.74	8.23	0.84	0.27^d^	1.16
	Coastal 4 N – 40 NaCl	31.2	2.35	8.46	0.82	0.27^d^	1.18
	Coastal 16 N – 0.3 NaCl	26.7	2.85	8.01	0.94	0.33^c^	1.29
	Coastal 16 N – 20 NaCl	33.2	2.70	8.17	0.90	0.31^cd^	1.30
	Coastal 16 N – 40 NaCl	29.1	2.69	8.14	0.99	0.33^c^	1.37
P values	Ecotype (E)	0.0004	0.0295	0.0000	0.0000	0.0000	0.0000
	N level (N)	0.6941	0.1501	0.5817	0.0004	0.0000	0.0001
	Salinity (S)	0.2090	0.0426	0.7572	0.0452	0.1825	0.0084
	E × N	0.5904	0.7559	0.6176	0.6812	0.8195	0.9026
	E × S	0.0186	0.1628	0.9861	0.0017	0.0074	0.0031
	N × S	0.3366	0.4204	0.6457	0.0042	0.0105	0.0071
	E × N × S	0.6789	0.6992	0.6510	0.3677	0.0433	0.2385

For each parameter, means for each salinity level (n = 4) within columns followed by different capital, lower-case, and tones letters are significantly different according to the Duncan’s multiple range test.

Although significant interactions were observed, the leaf of the montane ecotype exhibited higher chlorophyll a and b and total chlorophyll concentrations compared to the coastal-marine ecotype (**Table 2**). A significant E × S interaction was observed for chlorophyll a and total chlorophyll. In the montane ecotype, these parameters increased under both saline conditions (20 and 40 mM), while no difference was observed for the coastal-marine ecotype. Regardless of the ecotype, the leaf chlorophyll a and total chlorophyll were significantly higher when plants were grown under high N supply level combined with high salinity (20 and 40 mM) when compared with low-N treatments. At moderate and high salinity (20 and 40 mM NaCl), the leaf chlorophyll b in leaves of the montane ecotype was significantly increased by the higher total N supply level, compared with the other treatments.

### Mineral Composition and Partitioning

The increase of the external NaCl concentration to 20 mM raised the Cl^−^ concentration in the leaves of both stamnagathi ecotypes, while a further increase to 40 mM increased the leaf Cl^−^ only in the montane ecotype (**Table 3**). However, increasing the N concentration in the nutrient solution from 4 to 16 mM significantly reduced the leaf Cl^−^ concentration in the leaves of both ecotypes. Moreover, the leaf Cl^−^ concentration in the coastal-marine ecotype was always significantly higher than that in the montane ecotype within each NaCl and total-N level. The leaf Na^+^ concentration (**Table 3**) increased as the NaCl concentration in the supplied nutrient solution was raised from 0 to 40 mM in both ecotypes. However, at the highest NaCl salinity level (40 mM), the leaf Na^+^ concentration increased to higher levels in the montane than in the coastal-marine ecotype.

**Table 3:** Impact of seed origin (montane or coastal-marine ecotype), total nitrogen concentration (4 or16 mmol L^−1^, denoted as 4-N and 16-N, respectively), and salinity (0.3, 20, and 40 mM, respectively) in the nutrient solution supplied to hydroponically grown stamnagathi on leaf Cl, Na, K, Ca, Mg, P, organic N, and sulfates.

Sources of variation		Leaf Cl (mg g^−1^ DW) 1^st^ growing cycle	Leaf Na (mg g^−1^ DW) 1^st^ growing cycle	Leaf K (mg g^−1^ DW) 1^st^ growing cycle	Leaf Ca (mg g^−1^ DW) 1^st^ growing cycle	Leaf Mg (mg g^−1^ DW) 1^st^ growing cycle	Leaf P (mg g^−1^ DW) 1^st^ growing cycle	Leaf organic N (mg g^−1^ DW) 1^st^ growing cycle	Leaf sulfates (mg g^−1^ DW) 1^st^ growing cycle
Ecotype	Montane	25.6	12.6	38.4	9.2	1.70	8.07	38.3	3.00
	Coastal	38.4	11.6	52.8	15.3	2.48	7.87	34.2	3.17
N level (N)	4 mM	40.5	12.1	43.8	11.2	2.00	7.88	35.6	4.62
	16 mM	24.5	12.1	47.6	13.3	2.18	8.05	36.9	1.55
Salinity	0.3 mM	16.2	3.2^C^	53.0	13.7	2.38^a^	7.28^b^	36.8	3.34^A^
	20 mM	35.0	14.5^B^	46.2	12.7	2.08^b^	7.67^b^	35.5	2.91^B^
	40 mM	46.3	18.6^A^	37.8	10.3	1.82^c^	8.94^a^	36.5	3.01^AB^
**Interactions**									
E × N	Montane 4-N	31.3^B^	13.2	38.2	8.5	1.63	8.07	37.9	4.42
	Montane 16-N	20.0^C^	13.5	38.5	9.8	1.76	8.06	38.8	1.57
	Coastal 4-N	47.8^A^	11.9	49.2	13.9	2.37	7.69	33.3	4.81
	Coastal 16-N	29.0^B^	11.7	52.4	16.7	2.57	8.04	35.1	1.53
E × S	Montane 0.3 NaCl	11.9^e^	2.9^d^	47.3	10.6	1.95	7.30	37.3^a^	3.18
	Montane 20 NaCl	26.1^c^	15.5^c^	37.2	9.3	1.64	7.72	38.7^a^	2.89
	Montane 40 NaCl	38.8^b^	21.5^a^	30.5	7.6	1.51	9.19	39.0^a^	2.92
	Coastal 0.3 NaCl	20.5^d^	2.9^d^	58.4	16.9	2.81	7.25	36.3^ab^	3.49
	Coastal 20 NaCl	43.8^a^	14.8^c^	49.1	16.1	2.47	7.66	32.3^c^	2.93
	Coastal 40 NaCl	50.9^a^	17.7^b^	44.9	13.1	2.12	8.69	33.9^bc^	3.09
N × S	4 N – 0.3 NaCl	23.1	2.9	51.1	12.2	2.37	7.25	34.5^B^	5.15^a^
	4 N – 20 NaCl	40.5	15.2	43.0	11.0	1.86	7.99	34.7^B^	4.28^c^
	4 N – 40 NaCl	57.8	19.6	37.0	10.5	1.78	8.40	37.5^AB^	4.42^b^
	16 N – 0.3 NaCl	9.3	3.0	54.6	15.3	2.39	7.30	39.1^A^	1.52^d^
	16 N – 20 NaCl	29.4	15.1	43.3	14.4	2.25	7.38	36.3^AB^	1.54^d^
	16 N – 40 NaCl	34.8	19.6	38.4	10.1	1.85	9.48	35.4^B^	1.59^d^
E × N × S	Montane 4 N – 0.3 NaCl	18.0	2.8	48.7^bc^	9.9^e^	1.97	7.79	34.2	4.79
	Montane 4 N – 20 NaCl	28.2	15.3	33.8^e^	8.0^f^	1.48	7.86	37.6	4.22
	Montane 4 N – 40 NaCl	47.2	21.5	32.1^e^	7.6^f^	1.46	8.57	41.8	4.25
	Montane 16 N – 0.3 NaCl	5.7	3.1	45.9^cd^	11.2^de^	1.94	6.81	40.4	1.56
	Montane 16 N – 20 NaCl	24.0	15.6	40.6^d^	10.6^e^	1.81	7.57	39.8	1.56
	Montane 16 N – 40 NaCl	30.3	21.6	28.9^e^	7.6^f^	1.55	9.80	36.2	1.59
	Coastal 4 N – 0.3 NaCl	28.1	3.1	53.4^b^	14.4^b^	2.77	6.71	34.8	5.50
	Coastal 4 N – 20 NaCl	52.8	15.1	52.3^b^	14.0^bc^	2.25	8.12	31.8	4.34
	Coastal 4 N – 40 NaCl	62.4	17.8	41.9^d^	13.6^bc^	2.09	8.23	33.2	4.59
	Coastal 16 N – 0.3 NaCl	12.9	2.9	63.4^a^	19.4^a^	2.85	7.78	37.8	1.48
	Coastal 16 N – 20 NaCl	34.7	14.5	58.0^a^	18.2^a^	2.70	7.19	32.8	1.51
	Coastal 16 N – 40 NaCl	39.3	17.6	48.0^bc^	12.6^cd^	2.16	9.15	34.6	1.59
P values	Ecotype (E)	0.0027	0.0386	0.0000	0.0000	0.0000	0.2927	0.0000	0.0788
	N level (N)	0.0000	0.9155	0.1121	0.0000	0.0290	0.3519	0.1241	0.0000
	Salinity (S)	0.0000	0.0000	0.0000	0.0000	0.0000	0.0029	0.4254	0.0456
	E × N	0.0168	0.3158	0.1810	0.0187	0.6192	0.1497	0.6397	0.0506
	E × S	0.0157	0.0090	0.4106	0.2178	0.3288	0.7593	0.0391	0.7172
	N × S	0.1316	0.9514	0.4676	0.0000	0.1050	0.8327	0.0128	0.0403
	E × N × S	0.4351	0.9794	0.0000	0.0092	0.9127	0.3192	0.0512	0.6479

For each parameter, means for each salinity level (n = 4) within columns followed by different capital and lower-case letters are significantly different according to the Duncan’s multiple range test.

The leaf K^+^ was significantly higher in the coastal-marine ecotype than in the montane ecotype when compared within each combination of high salinity with low and high N concentrations, as shown by the significant E × N × S interaction. However, the level of salinity that reduced the leaf K^+^ was depending on both the ecotype and the total-N supply level (no difference between both ecotypes was observed under low NaCl and low N). In particular, in plants of the montane ecotype exposed to low N-supply, the leaf K^+^ was significantly reduced by exposure to 20 mM NaCl, while exposure to 40 mM did not further reduce the leaf K^+^. On the other hand, under high N-supply, no difference was observed for both ecotypes exposed to 20 mM NaCl, while leaf K^+^ decrease was observed at 40 mM NaCl.

A significant E × N × S interaction was observed for the leaf Ca^2+^ concentration. The leaf Ca^2+^ was significantly higher in the coastal-marine ecotype than in the montane ecotype, when compared within each combination of salinity and total-N supply, except for coastal ecotype exposure to 16N-40 NaCl, which was similar to montane 16N-0.3 NaCl. High N supply (16 mM) decreased significantly the leaf Ca^2+^ at both ecotypes when exposed to high salinity (40 mM NaCl). However, at the high level of total-N supply, the leaf Ca^2+^ was significantly reduced only by exposure to 40 mM NaCl, while 20 mM NaCl had no significant impact on the leaf Ca^2+^ concentration. At the low level of total-N supply, the leaf Ca^2+^ was significantly reduced by exposure to 20 mM NaCl without any additional reduction when salinity was further increased to 40 mM NaCl in the montane ecotype, while it was not influenced by salinity in the coastal-marine ecotype.

The leaf Mg^2+^ was significantly higher in the coastal-marine ecotype than in the montane ecotype, regardless of salinity and total-N supply level. Furthermore, the high total-N supply level increased slightly but significantly the leaf Mg^2+^ concentration without any significant interaction with ecotype and salinity. Increasing the salinity from 0.3 to 20 mM NaCl in the supplied nutrient solution decreased significantly the leaf Mg^2+^, while a further increase of salinity to 40 mM NaCl reduced the leaf Mg^2+^ to even lower levels, without any interaction with the ecotype and the level of N-supply.

The increase of salinity from 0.3 to 40 mM NaCl of the supplied nutrient solution increased leaf phosphorus (P) concentration significantly regardless of the ecotype. At the lower total N supply level, exposure of stamnagathi to NaCl salinity reduced the leaf SO_4_^2−^ concentration regardless of the ecotype, while for the high N supply level no significant differences were found. For both ecotypes, increasing N-supply decreased leaf SO_4_^−2^ concentration. Under salinity conditions, the leaf organic N content was significantly greater in the montane ecotype compared to that measured in the coastal-marine ecotype and for material harvested from both production cycles (data shown only for the first production cycle), while no significant difference was observed at 0.3 mM NaCl (**Table 3**). A significant interaction between total-N and salinity indicated that under non-saline conditions a high total-N supply (16 mM) resulted in higher leaf organic-N levels compared to low N supply (4 mM).

The leaf Fe and Mn concentrations in the coastal-marine ecotype were significantly higher than in the montane ecotype, although significant interactions were observed for Mn (**Supplementary Table 2**). Salinity and N supply increased the leaf Fe concentration regardless of ecotype. The leaf Mn concentration of the montane ecotype was increased when the total N increased from 4 to 16 mM. The highest leaf Mn concentration was observed when plants were grown under no salinity combined with high total N level, compared to the other treatments. Similarly, an increase in the total N level from 4 to 16 mM increased the leaf Zn concentration of the montane ecotype, while the inverse was observed in the coastal-marine ecotype. The leaf Mn and Zn concentrations in the coastal-marine ecotype were reduced under moderate and high salinity conditions, while no differences were observed in the montane ecotype. However, salinity decreased the leaf Zn concentration under low total N, while under high total N, the leaf Zn was increased by salinity at 20 mM NaCl and decreased at 40 mM NaCl. Salinity increased the leaf Cu in the coastal-marine ecotype at both N supply levels, while in the montane ecotype salinity had no impact on leaf Cu.

The root Na^+^ concentration increased as the NaCl concentration in the supplied nutrient solution was raised from 0 to 40 mM, but the increase was depending on both the ecotype and the N-supply level as indicated by the significant ecotype × N-supply × salinity interaction (**Supplementary Table 1**). The root Na^+^ concentration was significantly higher in the montane than in the coastal-marine ecotype at all salinity levels but the differences were larger at the moderate (20 mM) and the high (40 mM) salinity levels.

The root K^+^ and Ca^2+^ concentrations were significantly higher in the montane ecotype compared to those measured in the coastal-marine ecotype, regardless of salinity and total-N supply level (**Supplementary Table 1**). The level of total-N supply had no significant impact on the root K^+^ and Ca^2+^ concentrations. Increasing the salinity from 0.3 to 20 or 40 mM NaCl in the supplied nutrient solution resulted in a significant decrease of the root K^+^ and Ca^2+^. The root Mg^2+^ concentration was not influenced by either of the treatments tested in the current study. The root P concentration was significantly higher in the montane population compared to that originating from a coastal-marine habitat. The organic-N concentration was significantly higher in the roots of the montane ecotype compared to the coastal-marine ecotype under non-saline conditions, but this difference disappeared when the plants of both ecotypes were exposed to 20 or 40 mM NaCl. The level of total-N supply and salinity had no consistent impact on the organic-N concentration in the roots of stamnagathi.

Root Fe, Zn, and Cu concentrations of montane ecotype were reduced under high salinity conditions regardless of the total N level applied. The root Fe, Mn, Zn, and Cu concentration in the coastal-marine ecotype increased by increasing the salinity from 0.3 to 40 mM NaCl under low N concentration, while the inverse was the case under high total N concentrations, except for Mn. Root Mn of the montane ecotype was not affected by the salinity level when the N supply was low (**Supplementary Table 2**).

### Bioactive Content and Radical Scavenging Activity

The leaves of the montane ecotype contained significantly more total phenols (61%), carotenoids (42%), and flavonoids (28%) than the coastal-marine ecotype, although significant interactions were observed (**Table 4**). Furthermore, the montane ecotype exhibited a significantly higher antioxidant activity than the coastal-marine ecotype under low salinity. Salinity increased significantly the total phenolics concentration in the leaves of both ecotypes, regardless of the N-supply level (**Table 4**). However, in the coastal-marine ecotype, the increase of total N supply had no impact on total phenolics, while it reduced its content for the montane ecotype.

**Table 4:** Impact of seed origin (montane or coastal-marine ecotype), total nitrogen concentration (4 or 16 mmol L^−1^, denoted as 4-N and 16-N, respectively), and salinity (0.3, 20, and 40 mM, respectively) in the nutrient solution supplied to hydroponically grown stamnagathi on total phenolics, flavonoids, carotenoids, and antioxidant activity.

Sources of variation		Total phenolics (mg GAE 100 g^−1^ FW) 1^st^ growing cycle	Flavonoids (mg catechin 100 g^−1^ FW) 1^st^ growing cycle	Carotenoids (mg 100 ml^−1^ extraction) 1^st^ growing cycle	Antioxidant activity (mg Trolox 100 g^−1^ FW) 1^st^ growing cycle
Ecotype	Montane	164.4	67.1	1.28	35.1
	Coastal	104.1	52.3	0.88	31.4
N level (N)	4 mM	136.8	58.7	1.09	33.2
	16 mM	131.7	60.7	1.07	33.4
Salinity	0.3 mM	114.9^B^	63.8	1.10^ab^	30.7
	20 mM	136.9^A^	57.9	1.17^a^	34.6
	40 mM	150.9^A^	57.4	0.98^b^	34.5
**Interactions**					
E × N	Montane 4-N	173.0^a^	64.8	1.28	34.7
	Montane 16-N	155.7^b^	69.3	1.27	35.5
	Coastal 4-N	100.6^c^	52.6	0.90	31.6
	Coastal 16-N	107.6^c^	52.0	0.86	31.2
E × S	Montane 0.3 NaCl	135.8	72.8	1.30	34.7
	Montane 20 NaCl	170.5	64.6	1.42	35.2
	Montane 40 NaCl	186.7	63.9	1.12	35.4
	Coastal 0.3 NaCl	94.0	54.8	0.89	26.7
	Coastal 20 NaCl	103.3	51.2	0.91	33.9
	Coastal 40 NaCl	115.1	50.9	0.85	33.7
N × S	4 N – 0.3 NaCl	112.1	60.9	1.12	31.6
	4 N – 20 NaCl	139.3	59.3	1.20	33.9
	4 N – 40 NaCl	159.1	56.0	0.96	34.0
	16 N – 0.3 NaCl	117.8	66.8	1.07	29.8
	16 N – 20 NaCl	134.5	56.5	1.13	35.2
	16 N – 40 NaCl	142.7	58.8	1.00	35.0
E × N × S	Montane 4 N – 0.3 NaCl	143.3	65.6^bc^	1.37	34.4^abc^
	Montane 4 N – 20 NaCl	183.6	67.0^b^	1.42	34.5^abc^
	Montane 4 N – 40 NaCl	192.0	61.9^de^	1.05	35.2^ab^
	Montane 16 N – 0.3 NaCl	128.3	80.0^a^	1.22	35.0^abc^
	Montane 16 N – 20 NaCl	157.4	62.1^cd^	1.42	36.0^a^
	Montane 16 N – 40 NaCl	181.5	65.8^bc^	1.18	35.6^a^
	Coastal 4 N – 0.3 NaCl	80.8	56.1^ef^	0.86	28.7^d^
	Coastal 4 N – 20 NaCl	94.9	51.6^fg^	0.98	33.3^bc^
	Coastal 4 N – 40 NaCl	126.2	50.0^g^	0.87	32.9^c^
	Coastal 16 N – 0.3 NaCl	107.3	53.5^fg^	0.92	24.7^e^
	Coastal 16 N – 20 NaCl	111.6	50.8^g^	0.83	34.4^abc^
	Coastal 16 N – 40 NaCl	104.0	51.7^fg^	0.82	34.4^abc^
P values	Ecotype (E)	0.0000	0.0000	0.0000	0.0000
	N level (N)	0.3657	0.0126	0.4206	0.6054
	Salinity (S)	0.0000	0.0000	0.0343	0.0000
	E × N	0.0377	0.0024	0.8617	0.0927
	E × S	0.0869	0.0640	0.3194	0.0000
	N × S	0.3080	0.0007	0.5275	0.0052
	E × N × S	0.0975	0.0001	0.4595	0.0132

For each parameter, means for each salinity level (n = 4) within columns followed by different capital and lower-case letters are significantly different according to the Duncan’s multiple range test.

For the montane ecotype, the flavonoids level was reduced by increasing the salinity from 0.3 to 40 mM NaCl under both N concentrations, while flavonoid levels of the coastal-marine ecotype were reduced only under the low N levels. However, the antioxidant activity of the montane ecotype was not affected either by the salinity level or by the total N level. On the other hand, elevated levels of salinity increased the antioxidant activity of the coastal-marine ecotype under both N-supply levels.

## Discussion

The present study has revealed that stamnagathi is appreciably tolerant plant to salinity, and that different ecotypes exhibit different degrees of salt tolerance, which may depend also on the level of salt normally experienced under the natural conditions to which they have adapted. Indeed, NaCl levels up to 40 mM did not reduce the shoot FW in the coastal-marine ecotype, while the shoot FW of the montane ecotype was significantly reduced at salinity level of 40 mM. Klados and Tzortzakis (2014), Ntatsi et al. (2017a), and Petropoulos et al. (2017) also reported on a high tolerance of stamnagathi to salinity. In specific, Ntatsi et al. (2017a) showed that the montane ecotype of stamnagathi is tolerant to 40 and 80 mmol L^−1^ NaCl or isosmotic solutions of CaCl_2_, Na_2_SO_4_, and KCl. Using metabolomics analysis, Ntatsi et al. (2017a) showed that a major mechanism conferring high salt tolerance to stamnagathi is efficient osmoprotection due mainly to increased levels of γ-aminobutyric acid (GABA), glutamate, pyroglutamate, L-proline, and sucrose. In the study of Ntatsi and co-workers (2017a) the salt tolerance of stamnagathi was not associated with reduced transport of Na^+^ and Cl^−^ ions to the photosynthetically active leaves. Combining these considerations and the metabolomics analysis, led to the conclusion that osmoprotection and/or efficient compartmentation of the toxic salt ions to the vacuoles is the major mechanism by which saline-tolerant genotypes of stamnagathi may combat salinity. Controlled salt inclusion combined with efficient intracellular compartmentation of the salt ions to the vacuoles appears as the most efficient strategy deployed by salt tolerant plant species to sustain plant growth rates in saline environments (Shabala and Cuin, 2007; Munns and Gilliham, 2015; Liang et al., 2018). As a rule, plants relying on salt exclusion to combat salinity, which retain more Na^+^ in the roots while transporting less Na^+^ and/or Cl^−^ to the photosynthetically active leaves, are vulnerable to salinity (Shabala and Cuin, 2007; Wu, 2018). In the current study, the coastal-marine ecotype, which exhibited a higher salt tolerance than the montane ecotype, contained appreciably more Cl^−^ (33% on average) than the montane ecotype within each NaCl-salinity level, which shows that salt tolerance in stamnagathi is not associated with Cl^−^ exclusion. However, compared to the montane ecotype, the coastal-marine ecotype also contained less Na^+^ in the roots (48% on average) at both salinity levels and less Na^+^ in the leaves (8% on average) at the highest salinity level (40 mM NaCl), which indicates that efficient Na^+^ exclusion may play also a complementary role in the salt tolerance of this ecotype.

Under salinity conditions, salt tolerant plant species can decrease their leaf water potential by compartmentalizing the toxic Na^+^ and Cl^−^ salts to the vacuoles and synthesizing compatible organic solutes in the cytosol to avoid cell dehydration (Shabala, 2013; Bassil and Blumwald, 2014). As a result, a sufficiently high-water potential gradient between leaf cells and the external solution can be maintained, despite the salinity-induced decrease in the external water potential (Shabala, 2013; Mansour and Ali, 2017). However, many compatible organic solutes contributing to osmoregulation between the cytosol and the vacuole bear fixed negatively charged sites, i.e., carboxylic anions, which are electrochemically balanced by K^+^ (Munns and Gilliham, 2015). Furthermore, Mg-ATP and Mg-PP_i_ provide energy to proton pumps transporting actively H^+^ into the vacuoles, which are subsequently excreted back to the cytosol through Na^+^/H^+^ antiporters, thereby facilitating Na^+^ sequestration to vacuoles (Roy et al., 2014). In agreement with this consideration, the K^+^ and Mg^2+^ concentrations in the leaves of the coastal-marine ecotype, which exhibited a higher tolerance to salinity, were significantly higher (27% and 31% respectively) than in leaves of the montane ecotype within each salinity and total-N supply level. Calcium contributes mainly to stabilization of the cell walls and membranes, while its concentration in the cytosol is low (Cabañero et al., 2006; Kader and Lindberg, 2010). However, calcium is associated with cell membrane protection against disruption caused by increased external NaCl salinity (Rengel, 1992; Tuna et al., 2007). Furthermore, Ca^2+^ and other divalent cations (Mg^2+^, Zn^2+^) may prevent K^+^ leakage in response to salinity, thus contributing to maintenance of a higher K^+^/Na^+^ ratio in the cytoplasm, and concomitantly to higher salt tolerance (Shabala and Cuin, 2007). Consequently, the higher leaf Ca^2+^ concentration in the coastal-marine ecotype within each salinity and total-N supply level is in-line with its higher salt tolerance compared to that of the montane ecotype.

The higher organic-N concentrations in the leaves of the montane ecotype when the plants were exposed to NaCl-salinity may be associated with the lower leaf Cl^−^ concentrations in this ecotype. Indeed, Cl^−^ acts antagonistically to the NO_3_^−^ uptake, and plants exposed to NaCl salinity show generally lower leaf NO_3_^−^ concentrations (Munns and Termaat, 1986; Knight, et al. 1992; Grattan and Grieve, 1998). A higher NO_3_^−^ availability in the leaves of the montane ecotype owing to lower leaf Cl^−^ levels resulted presumably in higher rates of NO_3_^−^ assimilation into organic N compounds.

Salinity has been reported to either increase, restrict, or have no impact on tissue phosphorus concentrations. Salinity-induced increases in the tissue P concentrations have been observed mainly in experiments with plants grown hydroponically (Grattan and Grieve, 1998). The P concentrations in nutrient solutions applied to hydroponic crops are far higher than those typically found in the soil solution. As suggested by Nieman and Clark (1976), P concentrations that are optimal under non-saline conditions may impose a higher P uptake to levels resulting even to toxic tissue P concentrations in crops grown hydroponically under saline conditions. Nevertheless, in the present study, the increase of tissue P at 40 mM NaCl due to salinity did not result in any visible symptoms of P-toxicity.

Our results on plant biomass production of stamnagathi at both total-N supply levels corroborate the finding of Chatzigianni et al. (2018). With respect to the interactions of the total-N supply with salinity treatment, our results showed that the NaCl salinity had no impact on leaf sulfates when the supply of total-N was 16 mM, but reduced the SO_4_^2−^ uptake when the total-N supply was lowered to 4 mM. It is well known that sulfates (SO_4_^2−^) compete with both nitrates and chlorides in uptake sites (Kinjo and Pratt, 1971; Marschner, 2012). Thus, it seems that, in the present study, the high NO_3_^−^ concentration suppressed any antagonistic effect of Cl^−^ on SO_4_^2−^ uptake, while at low total-N, which entailed low NO_3_^−^ levels in the root zone, increasing levels of Cl^−^ restricted the uptake of SO_4_^2−^. Another salinity × total-N interaction was that the high N supply resulted in higher leaf organic-N levels only in the absence of NaCl salinity. Presumably, this is because under saline conditions the restriction of NO_3_-uptake by Cl^−^ (Grattan and Grieve, 1998) counteracted the enhanced NO_3_-supply in the high-N treatment.

In contrast to the leaf Ca^2+^, the root Ca^2+^ concentration was significantly higher in the montane ecotype compared to that measured in the coastal-marine ecotype. A likely explanation for this finding is that the greater root Ca^2+^ concentration is linked with the higher root Na^+^ concentrations for the montane ecotype. It is well established that Ca^2+^ enhances membrane selectivity to ion uptake and transport through the plasma membrane and the tonoplast (Hepler, 2005). Thus, the greater root Na^+^ concentrations in the coastal-marine ecotype may have stimulated Ca^2+^ accumulation to the root cells aiming to a more-efficient compartmentation of root Na^+^ to the vacuoles of the root xylem parenchyma.

Of the four metallic micronutrients, only Fe exhibited a consistent tendency to increase its concentration in the leaf tissues with increasing salinity, regardless of the stamnagathi ecotype and the level of total-N supply. Increased tissue Fe concentrations with increasing salinity have been reported also by other investigators and for several plant species, including tomato, soybean, and squash (Mass et al., 1972), strawberry (Turhan and Eris, 2005), and zucchini squash (Víllora et al., 2000). However, in other studies dealing with different plant species, salinity had no effect on tissue-Fe level for lettuce (Lazof and Bernstein, 1999) and reduced it in sunflower (Sanchez-Raya and Delgado, 1996).

The impact of salinity on the concentrations of the other three metallic micronutrients was dependent upon the N-supply level and also stamnagathi ecotype. Mass et al. (1972), Víllora et al. (2000), Tunçturk et al. (2008), and Weisany et al. (2014) also found that the impact of salinity on the leaf and root concentrations of Mn and Zn in tomato, soybean, and squash was also dependent on the plant species and cultivar. These results indicate a link to the specific genotype, which dictates the mode and extent of salt tolerance deployed by each species, ecotype, and even cultivar. This is presumably the case with Cu, given that in the present study salinity raised considerably the Cu levels in leaves of the coastal-marine ecotype, which is more tolerant to salinity, while it had no impact (at 4 mM total-N) or imposed only a slight Cu increase (at 16 mM total-N) in the montane ecotype. All metallic macronutrients are constituents of antioxidant enzymes and/or other metabolites (Marschner, 2012). Thus, the strong differences in the leaf Fe, Mn, Zn, and Cu concentrations between the coastal-marine and the montane ecotype may be associated with commensurate differences in metabolic functions. The parameters measured in the research reported here do not allow for a clear understanding of the different mechanisms, which appear tο underpin the distinction of metabolic functions for the two ecotypes tested. Further research is needed to determine the metabolic profiles of the two ecotypes, which could be linked with the results of the mineral composition presented here.

Soilless culture systems are known to facilitate the precise application of *eustress* (positive stress) such as mild to moderate NaCl and nutritional (e.g., N) stress, through a precise management of the NS concentration, thus triggering the biosynthesis of secondary metabolites (phenols, flavonoids, carotenoids) necessary for adaptation to suboptimal environments (Rouphael and Kyriacou, 2018; Rouphael et al., 2018c). These antioxidant molecules are of high importance to human health and longevity, and thus, they indirectly attribute an extra value (compared to non-stressed crops) to the basic nutritional characteristics of leafy and fruit vegetables (Orsini et al., 2016).

Phenolic compounds act as co-substrates, decomposing H_2_O_2_ through oxidation by peroxidase, thereby contributing to ROS scavenging and thus to enhanced tolerance to oxidative stress (Sudhakar, et al., 2001). The slight increase of total phenolics as the salt level increased indicates that this large group of secondary metabolites contains antioxidant compounds that may well contribute to salt tolerance in stamnagathi. Lim et al. (2012) found that the exposure of buckwheat (*Fagopyrum esculentum* M.) sprouts to salinity enhanced the total phenolics content due mainly to an increase in the concentration of four compounds, particularly isoorientin, orientin, rutin, and vitexin. In contrast to the total phenolics, carotenoids and flavonoids (a sub-group of phenolics) did not increase when stamnagathi was exposed to salinity, which indicates that these antioxidant compounds are not linked with salt tolerance mechanisms in stamnagathi. In contrast to stamnagathi, other plant species exhibited an increase in both carotenoids and flavonoids under salt stress conditions (Lim et al., 2012; Wang et al., 2018). There were significantly greater concentrations of total phenolics, carotenoids, and flavonoids in the montane compared with the coastal-marine ecotype, irrespective of salinity exposure and level. Nevertheless, the coastal-marine ecotype was more efficient than the montane ecotype in increasing its antioxidant activity when the plants were facing salt-stress, and this may be associated with the naturally higher salt tolerance capacity of the coastal-marine ecotype. The higher concentrations of total phenolics carotenoids and flavonoids in the montane ecotype may be associated with enhanced ability of this ecotype to combat other stresses beyond salinity (e.g., low temperature stress). Indeed, recently published results have shown that the degree of low-temperature tolerance in grapevine varieties was positively related with their total phenolics content (Król et al., 2015).

In the present study, the leaf chlorophyll content was influenced more by ecotype than by salinity or the level of N supply. Petropoulos et al. (2016) also reported different leaf chlorophyll concentrations between different ecotypes of *C. spinosum*. The higher leaf chlorophyll concentrations in the less salt tolerant montane ecotype, and similar leaf chlorophyll concentrations at all salinity levels in the coastal-marine ecotype, show that the biosynthesis of this pigment plays a key role in stamnagathi salt tolerance. Thus, the higher leaf chlorophyll concentrations of the montane ecotype may be related to higher tolerance to another abiotic stress. Given that during its evolution the montane ecotype was faced with much lower temperatures during the winter than the coastal-marine ecotype, the higher chlorophyll concentration in this ecotype may be an adaptation aiming to enhance its tolerance to cold. Indeed, as reported by Sanghera et al. (2011) and Li et al. (2018), higher leaf chlorophyll concentrations in plants are associated with higher cold tolerance.

The higher dry matter content in the montane ecotype may also be related to a higher cold tolerance for this ecotype compared to that originating from a coastal location. Indeed, a higher tolerance of plants to sub-optimal temperatures is associated with a higher dry matter percentage originating mainly from increased starch accumulation (Venema et al., 1999; Król et al., 2015; Ntatsi et al., 2017b). On the other hand, a low dry matter content in leaves may be a consequence of increased leaf succulence, which is an adaptive morphological characteristic of plants to salinity (Greenway and Munns, 1980; Shabala, 2013). Thus, an additional explanation for the lower dry matter content in the coastal-marine ecotype is that these plants tend to develop leaves that are more succulent as an adaptation to salinity, which agrees with its higher salt tolerance compared to that of the montane ecotype.

## Conclusion

A general conclusion derived from this study is that the coastal-marine ecotype is superior in terms of salt tolerance compared to the montane ecotype and can be used as a good source of germplasm in breeding programs aiming to develop stamnagathi cultivars, which are resilient to salinity stress. On the other hand, the montane ecotype is superior in terms of nutritional value, and it presents leaves with better texture (more crispy) due to the appreciably higher dry matter content of their edible shoots, and it contains more total phenols, flavonoids, and carotenoids. Also, under non-saline conditions, the montane ecotype has a higher antioxidant capacity. An additional trait of the montane ecotype, which advocates for a higher nutritional quality compared to the coastal-marine ecotype, is its lower nitrate concentration in the edible shoot (Chatzigianni et al., 2018), especially when the N supply is not low.

## Author Contributions

Conceived and designed the experiments: DS and IL. Performed the experiments and the analyses: MC, MT, AS, IL, GN, and DS. Analyzed the data: MC, GN, YR, MT, and DS. Wrote the paper: MC, GN, YR, and DS. Reviewed the paper: GN, IL, YR, and DS. All authors have read and approved the manuscript.

## Funding

During the 3 years of experiments, candidate PhD student MC was supported by a scholarship from Bodossakis Foundation.

## Conflict of Interest Statement

The authors declare that the research was conducted in the absence of any commercial or financial relationships that could be construed as a potential conflict of interest.
