# Supplementary material for: Functional Quality, Mineral Composition and Biomass Production in Hydroponic Spiny Chicory (Cichorium spinosum L.) Are Modulated Interactively by Ecotype, Salinity and Nitrogen Supply
Source: Front Plant Sci. 2019 Aug 30;10:1040. doi: 10.3389/fpls.2019.01040 (PMC6727868; doi:10.3389/fpls.2019.01040)
Supplement: Supplementary file 1 [file DataSheet_1.docx]

Supplementary Material

**Supplementary table 1:** Impact of seed origin (montane or coastal-marine ecotype), total nitrogen concentration (4 or16 mmol L^-1^, denoted as 4-N and 16-N, respectively) and salinity (0.3 mM, 20 mM and 40 mM, respectively) in the nutrient solution supplied to hydroponically-grown stamnagathi on root Na, K, Ca, Mg, P and organic N.

| Sources of variation |  | Root Na (mg g^-1^ DW)  2^nd^ growing cycle | Root K  (mg g^-1^ DW)  2^nd^ growing cycle | Root Ca (mg g^-1^ DW)  2^nd^ growing cycle | Root Mg (mg g^-1^ DW)  2^nd^ growing cycle | Root P  (mg g^-1^ DW)  2^nd^ growing cycle | Root organic N (mg g^-1^ DW)  2^nd^ growing cycle |
| --- | --- | --- | --- | --- | --- | --- | --- |
| Ecotype | Montane | 7.76 | 22.6 | 3.78 | 1.10 | 5.85 | 2.13 |
|  | Coastal | 4.04 | 19.2 | 3.00 | 1.08 | 4.99 | 1.88 |
| N level (N) | 4 mM | 6.28 | 21.0 | 3.50 | 1.11 | 5.62 | 2.01 |
|  | 16 mM | 5.42 | 20.6 | 3.28 | 1.07 | 5.22 | 2.00 |
| Salinity | 0.3 mM | 2.90 | 24.8 ^a^ | 3.84 ^a^ | 1.11 | 5.41 | 1.87 |
|  | 20 mM | 6.52 | 20.0 ^b^ | 3.30 ^b^ | 1.05 | 5.46 | 2.11 |
|  | 40 mM | 8.14 | 17.8 ^b^ | 3.04 ^b^ | 1.11 | 5.39 | 2.05 |
| Interactions | | | | | | | |
| E x N | Montane 4-N | 8.33 | 22.8 | 3.99 | 1.15 | 6.17 | 2.16 |
|  | Montane 16-N | 7.20 | 22.2 | 3.58 | 1.05 | 5.53 | 2.10 |
|  | Coastal 4-N | 4.24 | 19.1 | 3.00 | 1.07 | 5.06 | 1.85 |
|  | Coastal 16-N | 3.64 | 19.1 | 2.98 | 1.09 | 4.92 | 1.90 |
| E x S | Montane 0.3 NaCl | 3.64 | 28.0 | 4.41 | 1.09 | 5.95 | 2.23 ^a^ |
|  | Montane 20 NaCl | 9.39 | 21.6 | 3.66 | 1.12 | 5.78 | 2.11 ^a^ |
|  | Montane 40 NaCl | 10.27 | 18.0 | 3.28 | 1.09 | 5.82 | 2.06 ^a^ |
|  | Coastal 0.3 NaCl | 2.15 | 21.6 | 3.25 | 1.04 | 4.87 | 1.50 ^b^ |
|  | Coastal 20 NaCl | 3.65 | 18.4 | 2.94 | 1.10 | 5.15 | 2.10 ^a^ |
|  | Coastal 40 NaCl | 6.01 | 174 | 2.78 | 1.10 | 4.96 | 2.03 ^a^ |
| N x S | 4 N – 0.3 NaCl | 2.93 | 24.2 | 4.02 | 1.12 | 5.57 | 1.89 ^BC^ |
|  | 4 N – 20 NaCl | 7.08 | 20.2 | 3.40 | 1.10 | 5.70 | 1.94 ^BC^ |
|  | 4 N – 40 NaCl | 8.84 | 18.6 | 3.06 | 1.11 | 5.58 | 2.19 ^AB^ |
|  | 16 N – 0.3 NaCl | 2.86 | 25.5 | 3.64 | 1.00 | 5.25 | 1.84 ^C^ |
|  | 16 N – 20 NaCl | 5.97 | 19.8 | 3.20 | 1.12 | 5.22 | 2.27 ^A^ |
|  | 16 N – 40 NaCl | 7.44 | 16.8 | 3.00 | 1.09 | 5.20 | 1.90 ^BC^ |
| E x N x S | Montane 4 N – 0.3 NaCl | 3.56 ^f^ | 26.1 | 4.68 | 1.15 | 6.10 | 2.26 |
|  | Montane 4 N – 20 NaCl | 10.58 ^a^ | 23.1 | 3.94 | 1.17 | 6.46 | 2.09 |
|  | Montane 4 N – 40 NaCl | 10.83 ^a^ | 19.3 | 3.32 | 1.13 | 5.94 | 2.14 |
|  | Montane 16 N – 0.3 NaCl | 3.71 ^f^ | 29.9 | 4.14 | 1.03 | 5.80 | 2.20 |
|  | Montane 16 N – 20 NaCl | 8.20 ^c^ | 20.1 | 3.35 | 1.06 | 5.09 | 2.13 |
|  | Montane 16 N – 40 NaCl | 9.70 ^b^ | 16.7 | 3.24 | 1.06 | 5.69 | 1.98 |
|  | Coastal 4 N – 0.3 NaCl | 2.29 ^g^ | 22.2 | 3.37 | 1.10 | 5.03 | 1.52 |
|  | Coastal 4 N – 20 NaCl | 3.58 ^f^ | 17.2 | 2.85 | 1.02 | 4.94 | 1.79 |
|  | Coastal 4 N – 40 NaCl | 6.84 ^d^ | 18.0 | 2.79 | 1.09 | 5.21 | 2.24 |
|  | Coastal 16 N – 0.3 NaCl | 2.00 ^g^ | 21.1 | 3.14 | 0.98 | 4.70 | 1.48 |
|  | Coastal 16 N – 20 NaCl | 3.73 ^f^ | 19.5 | 3.04 | 1.18 | 5.35 | 2.40 |
|  | Coastal 16 N – 40 NaCl | 5.18 ^e^ | 16.8 | 2.76 | 1.12 | 4.70 | 1.82 |
| P values | Ecotype (E) | 0.0000 | 0.0015 | 0.0000 | 0.6022 | 0.0015 | 0.0021 |
|  | N level (N) | 0.0000 | 0.7541 | 0.1425 | 0.3651 | 0.1310 | 0.8848 |
|  | Salinity (S) | 0.0000 | 0.0000 | 0.0002 | 0.6371 | 0.9848 | 0.0613 |
|  | E x N | 0.1349 | 0.7610 | 0.1853 | 0.1470 | 0.3466 | 0.5321 |
|  | E x S | 0.0000 | 0.0700 | 0.1865 | 0.8263 | 0.6842 | 0.0010 |
|  | N x S | 0.0077 | 0.4332 | 0.6585 | 0.3467 | 0.9831 | 0.0165 |
|  | E x N x S | 0.0008 | 0.1106 | 0.5702 | 0.4108 | 0.2252 | 0.1303 |

*For each parameter, means for each salinity level (n = 4) within columns followed by different capital and lower-case letters respectively are significantly different according to the Duncan’s multiple range test.*

**Supplementary table 2:** Impact of seed origin (montane or coastal-marine ecotype), total nitrogen concentration (4 or16 mmol L^-1^, denoted as 4-N and 16-N, respectively) and salinity (0.3 mM, 20 mM and 40 mM, respectively) in the nutrient solution supplied to hydroponically-grown stamnagathi on leaf and root Fe, Mn, Zn and Cu.

| Sources of variation |  | Leaf Fe (mg kg^-1^ DW)  2^nd^ growing cycle | Leaf Mn (mg kg^-1^ DW)  2^nd^ growing cycle | Leaf Zn (mg kg^-1^ DW)  2^nd^ growing cycle | Leaf Cu (mg kg^-1^ DW)  2^nd^ growing cycle | Root Fe (mg kg^-1^ DW)  2^nd^ growing cycle | Root Mn (mg kg^-1^ DW)  2^nd^ growing cycle | Root Zn (mg kg^-1^ DW)  2^nd^ growing cycle | Root Cu (mg kg^-1^ DW)  2^nd^ growing cycle |
| --- | --- | --- | --- | --- | --- | --- | --- | --- | --- |
| Ecotype | Montane | 66.21 | 29.4 | 68.0 | 5.57 | 29.1 | 18.6 | 69.9 | 10.5 |
|  | Coastal | 97.75 | 58.1 | 94.0 | 9.48 | 28.9 | 16.2 | 80.3 | 11.5 |
| N level (N) | 4 mM | 77.45 | 41.9 | 83.4 | 7.79 | 30.8 | 18.8 | 80.4 | 10.9 |
|  | 16 mM | 86.52 | 45.6 | 78.5 | 7.26 | 27.2 | 15.9 | 69.7 | 11.2 |
| Salinity | 0.3 mM | 73.38 ^b^ | 50.2 | 87.9 | 5.90 | 31.6 | 16.7 | 85.7 | 13.1 |
|  | 20 mM | 80.02 ^a^ | 41.8 | 83.2 | 8.17 | 29.3 | 18.4 | 67.9 | 10.0 |
|  | 40 mM | 92.55 ^a^ | 39.4 | 71.7 | 8.50 | 26.2 | 17.2 | 71.6 | 10.1 |
| Interactions | | | | | | | | | |
| E x N | Montane 4-N | 59.02 | 24.3 ^C^ | 61.5 ^C^ | 6.29 | 31.0 | 19.8 | 68.3 | 10.5 |
|  | Montane 16-N | 73.40 | 34.5 ^B^ | 74.4 ^B^ | 4.84 | 27.1 | 17.3 | 71.4 | 10.5 |
|  | Coastal 4-N | 95.87 | 59.5 ^A^ | 105.3 ^A^ | 9.28 | 30.5 | 17.8 | 92.5 | 11.2 |
|  | Coastal 16-N | 99.64 | 56.6 ^A^ | 82.6 ^B^ | 9.67 | 27.2 | 14.5 | 68.0 | 11.8 |
| E x S | Montane 0.3 NaCl | 53.34 | 28.1 ^c^ | 61.9 ^c^ | 5.36 | 35.0 | 19.3 | 87.5 | 13.6 |
|  | Montane 20 NaCl | 67.97 | 28.6 ^c^ | 72.2 ^c^ | 5.29 | 28.1 | 18.9 | 68.7 | 9.0 |
|  | Montane 40 NaCl | 77.33 | 31.6 ^c^ | 69.8 ^c^ | 6.06 | 24.3 | 17.5 | 53.4 | 8.9 |
|  | Coastal 0.3 NaCl | 87.80 | 72.2 ^a^ | 113.9 ^a^ | 6.44 | 28.2 | 13.9 | 83.9 | 12.5 |
|  | Coastal 20 NaCl | 99.72 | 55.0 ^b^ | 94.3 ^b^ | 11.05 | 30.4 | 17.8 | 67.1 | 10.9 |
|  | Coastal 40 NaCl | 105.73 | 47.1 ^b^ | 73.6 ^c^ | 10.94 | 28.0 | 16.8 | 89.8 | 11.2 |
| N x S | 4 N – 0.3 NaCl | 66.68 | 46.3 ^b′^ | 91.8 ^a′^ | 6.15 | 32.8 | 17.6 | 77.9 | 11.7 |
|  | 4 N – 20 NaCl | 80.08 | 37.3 ^b′^ | 75.7 ^b′^ | 8.32 | 30.1 | 19.1 | 73.9 | 10.0 |
|  | 4 N – 40 NaCl | 85.57 | 42.1 ^b′^ | 82.7 ^b′^ | 8.89 | 29.5 | 19.8 | 89.4 | 11.0 |
|  | 16 N – 0.3 NaCl | 74.46 | 54.0 ^a′^ | 84.0 ^b′^ | 5.65 | 30.4 | 15.7 | 93.4 | 14.4 |
|  | 16 N – 20 NaCl | 87.61 | 46.2 ^b′^ | 90.8 ^a′^ | 8.02 | 28.4 | 17.6 | 61.9 | 9.9 |
|  | 16 N – 40 NaCl | 97.49 | 36.6 ^b′^ | 60.7 ^c′^ | 8.11 | 22.8 | 14.5 | 53.8 | 9.2 |
| E x N x S | Montane 4 N – 0.3 NaCl | 47.1 | 18.9 | 53.2 | 6.52 ^bc^ | 38.3 ^a^ | 19.3 ^ab^ | 86.0 ^c^ | 13.1 ^ab^ |
|  | Montane 4 N – 20 NaCl | 60.8 | 23.8 | 58.0 | 5.74 ^bcd^ | 29.2 ^bcd^ | 20.7 ^a^ | 65.6 ^d^ | 9.7 ^cd^ |
|  | Montane 4 N – 40 NaCl | 69.1 | 30.2 | 73.2 | 6.61 ^bc^ | 25.6 ^de^ | 19.4 ^ab^ | 53.2 ^e^ | 8.7 ^e^ |
|  | Montane 16 N – 0.3 NaCl | 59.8 | 37.2 | 70.5 | 4.19 ^e^ | 31.6 ^bc^ | 19.3 ^ab^ | 88.9 ^c^ | 14.0 ^a^ |
|  | Montane 16 N – 20 NaCl | 75.1 | 33.3 | 86.4 | 4.83 ^de^ | 26.9 ^cde^ | 17.0 ^abc^ | 71.7 ^d^ | 8.3 ^e^ |
|  | Montane 16 N – 40 NaCl | 85.2 | 33.0 | 66.3 | 5.51 ^cde^ | 22.9 ^e^ | 15.6 ^cd^ | 53.5 ^e^ | 9.1 ^cd^ |
|  | Coastal 4 N – 0.3 NaCl | 86.3 | 73.7 | 130.3 | 5.77 ^bcd^ | 27.2 ^cde^ | 15.8 ^bcd^ | 69.8 ^d^ | 10.2 ^cd^ |
|  | Coastal 4 N – 20 NaCl | 99.3 | 50.8 | 93.4 | 10.90 ^a^ | 30.9 ^bcd^ | 17.4 ^abc^ | 82.1 ^c^ | 10.2 ^cd^ |
|  | Coastal 4 N – 40 NaCl | 102.0 | 54.0 | 92.1 | 11.17 ^a^ | 33.3 ^b^ | 20.1 ^a^ | 125.6 ^a^ | 13.2 ^ab^ |
|  | Coastal 16 N – 0.3 NaCl | 89.3 | 70.7 | 97.5 | 7.11 ^b^ | 29.1 ^bcd^ | 12.0 ^e^ | 97.9 ^b^ | 14.8 ^a^ |
|  | Coastal 16 N – 20 NaCl | 100.1 | 59.1 | 95.1 | 11.20 ^a^ | 29.9 ^bcd^ | 18.2 ^abc^ | 52.0 ^e^ | 11.5 ^bc^ |
|  | Coastal 16 N – 40 NaCl | 109.5 | 40.1 | 55.1 | 10.71 ^a^ | 22.6 ^e^ | 13.4 ^de^ | 54.0 ^e^ | 9.2 ^cd^ |
| P values | Ecotype (E) | 0.0000 | 0.0000 | 0.0000 | 0.0000 | 0.8068 | 0.0006 | 0.0000 | 0.0320 |
|  | N level (N) | 0.0402 | 0.1194 | 0.2304 | 0.0423 | 0.0006 | 0.0000 | 0.0000 | 05310 |
|  | Salinity (S) | 0.0014 | 0.0277 | 0.0075 | 0.0000 | 0.0001 | 0.0945 | 0.0000 | 0.0000 |
|  | E x N | 02313 | 0.0075 | 0.0001 | 0.3460 | 0.7059 | 0.5752 | 0.0000 | 0.4623 |
|  | E x S | 0.8851 | 0.0000 | 0.0001 | 0.0000 | 0.0001 | 0.0088 | 0.0000 | 0.0112 |
|  | N x S | 0.7759 | 0.0222 | 0.0027 | 0.0007 | 0.0679 | 0.0404 | 0.0000 | 0.0011 |
|  | E x N x S | 0.9109 | 0.2001 | 0.4471 | 0.0000 | 0.0037 | 0.0256 | 0.0000 | 0.0022 |

*For each parameter, means for each salinity level (n = 4) within columns followed by different capital, lower-case and tones letters respectively are significantly different according to the Duncan’s multiple range test.*
